# Passing the Baton: Substrate Channelling in Respiratory Metabolism

**DOI:** 10.1155/2018/1539325

**Published:** 2018-11-21

**Authors:** Alisdair R. Fernie, Youjun Zhang, Lee J. Sweetlove

**Affiliations:** ^1^Max-Planck-Institute of Molecular Plant Physiology, Am Mühlenberg 1, 14476 Potsdam-Golm, Germany; ^2^Center of Plant System Biology and Biotechnology, 4000 Plovdiv, Bulgaria; ^3^Department of Plant Sciences, University of Oxford, South Parks Road, Oxford OX1 3RB, UK

## Abstract

Despite species-specific differences in the pathways of respiratory metabolism are remarkably conserved across the kingdoms of life with glycolysis, the tricarboxylic acid cycle, and mitochondrial electron transport chain representing the major components of the process in the vast majority of organisms. In addition to being of critical importance in fueling life itself these pathways serve as interesting case studies for substrate channelling with research on this theme having been carried out for over 40 years. Here we provide a cross-kingdom review of the ample evidence for protein-protein interaction and enzyme assemblies within the three component pathways as well as describing the scarcer available evidence for substrate channelling itself.

## 1. Introduction

Respiratory metabolism consisting of the pathways of glycolysis, the TCA cycle, and the respiratory electron transport chain is a central feature of metabolic networks across all kingdoms of life, providing carbon skeletons for biosynthesis of a range of key metabolites, and is the heart of biochemical energy transformation. The basic chemistry and electrochemistry of the pathways are highly conserved [1, 2] with the possible exception of energy parasites such as diatoms and Chlamydiae [3, 4] and parasitic plants such as mistletoe that have reduced respiratory pathways [5, 6]. The core structures of each pathway are presented in Supplemental Figures [Sec supplementary-material-1]-[Sec supplementary-material-1]. Although the pathways function in different biochemical settings and are regulated accordingly, a degree of substrate channelling, the facilitated transfer of the metabolite product from one enzyme to the next enzyme in the pathway without that metabolite equilibrating with the bulk aqueous solvent [7], appears to be a common feature of these pathways across all kingdoms of life. This article will focus on this enigmatic phenomenon in respiratory metabolism, describing the nature of substrate channelling for each of the constituent pathways. Following a discussion of these pathways in isolation we will then provide an overview of their interaction as well as providing a perspective of the most important questions that remain for each pathway as well as for the general control of this fundamental biochemical process.

Rather than approaching respiratory pathways in the conventional metabolic order of (i) glycolysis, (ii) the TCA cycle, and (iii) mitochondrial electron transport chain [1, 8], we will lead with the pathway for which the most evidence for substrate channelling has been accrued, namely, the TCA cycle, before detailing glycolysis, and then finally the mitochondrial electron transport chain. The TCA cycle is one of the iconic pathways in metabolism being the first of a trinity of metabolic cycles elucidated by Hans Krebs in 1940 [9]. On the basis of his study in pigeon muscle the canonical view of the TCA cycle arose as a cycle of oxidation of respiratory substrates to drive ATP synthesis [10]. However, contemporary view of carboxylic acid metabolism has been expanded to a set of flux modes, of which full cyclic oxidation is only one, that are embedded in a larger metabolic network [11]. Some variation in the details of this pathway has been observed across nature [12]. However, the recent elucidation of the alternative route linking 2-oxoglutarate to succinyl–coenzyme A (CoA) [13] renders these differences more subtle than once thought. What is clear is that several different bypasses of the TCA cycle exist ranging from the near-ubiquitous GABA shunt to more taxonomically restricted bypasses such as acetate and maleate shunts [12]. These minor differences aside, the TCA cycle is remarkably conserved.

The pathway of glycolysis oxidizes glucose to pyruvate in a series of ten enzyme-catalyzed reactions concomitant with the production of ATP and NADH [14]. There are several variations of this pathway with the Embden-Meyerhof-Parnas (EMP) being the most prominent, but the Enter-Doudoroff and hetero- and homo-fermentative pathways are also present in a wide range of organisms [15]. The elucidation of these pathways took many years and as well as the pioneering achievements of Gustav Embden, Otto Meyerhof and Jakob Karol Parnas, rested on prior work carried out by, amongst others, Pasteur, Buchner, Harden, and Young [15]. There exist species-specific differences in the subcellular compartmentation of glycolysis with its constituent enzymes variously being reported as residing in the cytosol, plastid, and nucleus [16–18]. There are also differences in regulation with the plant enzymes, for example, not being subject to allosteric regulation by ATP [19]. However, as for the TCA cycle, the differences between glycolysis across the kingdoms of life are relatively small. Indeed it was recently demonstrated that plants are likely capable of carrying out the Enter-Doudoroff pathway of glycolysis [20], in which they were previously presumed to be incapable of supporting.

The final pathway of respiration, that of the mitochondrial electron transport chain, is arguably more divergent than the others with substantial differences in the sizes of the respiratory complexes and alternative respiratory pathways being present in plants that until recently had not been identified in microbial or mammalian systems [21]. It has, however, been studied for at least as long as glycolysis and longer than the TCA cycle with studies on this pathway being inspired by the work of Otto Warburg [22]. More recently, our understanding of the mechanistic function of the process has been enabled by molecular-level knowledge of the structures of the enzymes and electron transport complexes that comprise the respiratory chain and catalyze oxidative phosphorylation [22]. These studies led to our current understanding of the three proton-translocating complexes, Complexes I, III, and IV, and the mobile electron carriers ubiquinone and cytochrome* c* (see Supplemental [Sec supplementary-material-1]), and the mechanism of proton translocation to generate a proton-motive force for ATP synthesis is now well understood. While there are between-species differences in the exact composition of the respiratory complexes [23], as well as alternative pathways of respiration [24, 25], as for the other pathways of respiration, these are strongly outweighed by the commonalities.

## 2. Dynamic Enzyme Assemblies in the TCA Cycle

### 2.1. Early Observations of the Occurrence of Transient Enzyme Assemblies Involving Enzymes of the TCA Cycle

The earliest evidence for dynamic enzyme assemblies was reported over 40 years ago [26] although the assembly of individual polypeptide chains into multimeric proteins was realized considerably earlier [27]. The search for such dynamic assemblies was largely driven by the need to reconcile several kinetic observations that could not be explained by the conventional well-mixed metabolism. Specifically, isotope labelling experiments indicated no mixing of fatty acid- and pyruvate-derived acetyl-CoA, while the apparent concentration of free oxaloacetate was not sufficient to account for the rate of oxidation observed in the cycle [28]. Srere et al. further demonstrated that an immobilized pairing of malate dehydrogenase and citrate synthase had a kinetic advantage over the free enzymes [29]. A variety of other early experiments using a combination of electron microscopy, cross-linking, biochemistry, and calculations on the basis of stereomorphological measurements of mitochondria [26] further supported the concept of the organization of the TCA cycle enzymes that Srere termed the metabolon [30]. Since these pioneering studies, dynamic assemblies of consecutive enzymes have been observed across the kingdoms of life in a wide variety of pathways including glycolysis, oxidative phosphorylation, fatty acid, amino acid, polyketide, polyamine, and polypeptide biosynthesis [31, 32], with photosynthetic and natural product metabolons additionally being reported in plants [33, 34]. Moreover broad-scale screens in humans have suggested the presence of up to 130 000 binary protein-protein interactions at any one time [35, 36], underlining the commonality of such interactions.

While the early papers, mentioned above, were theoretically insightful, technical limitations mean that some of the conclusions look questionable with the benefit of hindsight. Nevertheless, the concept of the metabolon crystalized around these early studies and is now commonly held to define supramolecular complexes of sequential metabolic enzymes which tend to be noncovalently bound transient complexes allowing the regulation of metabolic pathway flux via association and/ or dissociation [7, 37, 38]. Furthermore, metabolons mediate substrate channelling (sometimes referred to as metabolite channelling), wherein reaction intermediates are isolated from the bulk solvent surrounding them.

**Figure 1:**
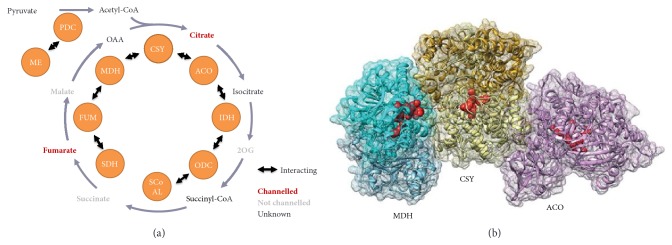
**The metabolon of the TCA cycle.** (a) The plant protein interactions of catalytic subunits that potentially mediate metabolite channelling are described next to the arrows. Metabolites drawn as red, grey, and black text are channelled, not channelled, and not tested, respectively [53]. (b) The protein structure of the metabolon MDH-CSY-ACO with the active sites (red spheres) [54, 55]. PDC, pyruvate dehydrogenase complex; ME, malic enzyme; CSY, citrate synthase; ACO, aconitase; IDH, isocitrate; ODC, oxoglutarate dehydrogenase complex; SCoAL, succinyl-CoA ligase; SDH, succinate dehydrogenase; FUM, fumarase; MDH, malate dehydrogenase; 2OG, 2-oxoglutarate.

Over a period of 25 years, studies using gel filtration and precipitation in polyethylene glycol revealed physical interactions between six of the eight sequential enzymes namely fumarase with malate dehydrogenase [39], malate dehydrogenase with citrate synthase [29], succinyl-CoA ligase with the oxoglutarate dehydrogenase complex [40], aconitase with citrate synthase [41] and isocitrate dehydrogenase with aconitase [42], and the oxoglutarate dehydrogenase complex [43]. These studies followed the observation that gentle sonication of isolated rat liver mitochondria yielded a preparation containing TCA cycle enzymes in a readily sedimentable form [43, 44]. Common features of all these studies is that they identified the interaction of enzymes by cosedimentation or cofiltration and assaying the enzyme couples revealed that they display a kinetic advantage in comparison to their activities in isolation. Considerable evidence has since accumulated that there are also interactions between TCA cycle enzymes and other proteins in the mitochondrial matrix and inner mitochondrial membrane [45–48] as well as members of the mitochondrial carrier family [49, 50]. However, it is important to note that the interaction of sequential enzymes (and even their aggregation in higher order assemblies) is not a proof of substrate channelling and there could be many other functions of such assemblies. But the clarity of the theoretical arguments made by Srere and Sugemi and their coworkers, backed up by a corroborating body of experimental support, cannot be underestimated in setting our contemporary views concerning enzyme interactions and substrate channelling and these researchers continued to make seminal contributions to our understanding of the TCA cycle metabolon. Two of those studies stand out. The first is a structural model of the malate-dehydrogenase-citrate synthase-aconitase complex [51] (Figure 1). Secondly, in a visionary experiment published in 1987, the degree of interaction between citrate synthase and malate dehydrogenase was quantified in the presence of various metabolites [52]. For this purpose a fluorescein isothiocyanate-labelled pig heart citrate synthase was incubated* in vitro* with malate dehydrogenase and a range of metabolites and the dissociation constant of the protein interactions were determined. Intriguingly, 2-oxoglutarate increased the dissociation constant while NADH lowered it. While the effect of NADH can easily be rationalized, since it is a major determinant of the energy-generating flux of the mitochondria, that of 2-oxoglutarate is more difficult to understand.

### 2.2. Application of Modern Systems-Based Approaches to Study Enzyme Assemblies of the TCA Cycle

More recently, comprehensive metabolic network-level characterization has been enabled by a combination of advances in fluorescence-based cell biology and proteomics. For example, a comprehensive characterization of the interactome between* Bacillus subtilis* enzymes revealed interactions between six consecutive enzymes of the TCA cycle (linking fumarate to succinyl-CoA), as well as interactions of these enzymes with phospho*enol*pyruvate carboxykinase and glutamate synthase [59]. Additionally, structural models of the TCA cycle enzyme complexes are consistent with substrate channelling via electrostatic retention of the channelled metabolite on charged domains of the enzyme surfaces [54, 55] (Figure 1(b)). Moreover, elegant studies wherein kinetics were probed by following diffusional motion of two sequential Krebs cycle enzymes in a microfluidic channel have demonstrated that the dynamics of protein association can be guided by cellular gradients of metabolite concentrations [60]. In addition to these studies using microbes or mammals, a comprehensive range of techniques including affinity purification mass spectrometry, split-luciferase, and yeast two-hybrid assays has been used to demonstrate protein-protein interactions in the plant TCA cycle [53]. This study also included an isotope dilution experiments which confirmed that citrate and fumarate are indeed channelled (these experiments are described in detail below) (Figure 1(a)).

One surprising feature of wide-scale protein interactome studies is the substantial number of interactions between nonconsecutive pathway enzymes as well as the expected interactions between consecutive enzymes. It is conceivable that many of these interactions, which occur largely between regulatory subunits of one enzyme and the catalytic subunit of another, acting as nucleation points which aid in the formation of metabolons [53] (Figure 1(a)). In a recent study, the extra-pathway interactions of the plant TCA cycle enzymes were revealed, with 125 interactions being identified that highlighted many novel interactions. One of these interactions was evaluated by analysis of knockout mutants of the mitochondrial glutaredoxin S15 and amidase in terms of their metabolite composition and metabolic flux profiles [61].

A survey of the biochemical literature suggests that the phenomenon of dynamic enzyme assembly is very common throughout nature both in the core high flux bearing pathways and in the specialized metabolism of plants and fungi [7, 30, 38]. The demonstration of protein-protein interaction in such assemblies is, however, often wrongly assumed to imply the presence of metabolons. In order to make such a claim, a far greater burden of proof must be met. Substrate channelling can occur by several different mechanisms: (i) metabolic intermediates may be covalently bound sequentially to active sites; (ii) in certain situations, such as that observed in the tryptophan synthase enzyme [62], a physical barrier within the enzyme complex forces noncovalently bound intermediates from one active site to another, (iii) site-to-site transfer of noncovalently bound intermediates, (iv) transfer of intermediates into an unstirred layer around the surface of the enzyme complex, or (iv) electrostatic effects mediating facilitated diffusion across the surface. In this section we will describe methods by which evidence for the occurrence of substrate channelling can be obtained and in each instance illustrate these with examples from the TCA cycle. The methods employed can be split into structural- and isotope-based approaches.

Recent advances in structural studies of the TCA cycle enzymes rely heavily on the earlier work of Srere and coworkers on the malate dehydrogenase-citrate synthase-aconitase complex, which we describe above. In a recent study, Wu and Minteer [54] coupled* in vivo* cross-linking and mass spectrometry to establish a low resolution structure of the malate dehydrogenase-citrate synthase-aconitase complex. Intriguingly, cross-linking revealed interactions between all eight enzymes of the TCA cycle. Using distance constraints derived from the cross-linking, two possible models of the malate dehydrogenase-citrate synthase-aconitase complex were proposed. In the first model, which is based on the most prevalent structure, the average distances between the active sites of the two enzymes are 35 angstrom and 50 angstrom for malate dehydrogenase-citrate synthase and aconitase-citrate synthase, respectively. A theoretical study by Elcock and McCammon [63] demonstrated that the presence of electrostatic interactions can greatly enhance substrate transport efficiency. Analysis of the modelled superstructure revealed that its surface regions are overrepresented by positively charged residues that provide a potential electrostatically defined passage between active sites for the negatively charged TCA cycle intermediates. Thus the enzyme complex potentially increases substrate transport efficiency between active sites, mediating substrate channelling. In a follow-up study, site-directed mutagenesis was performed on the highly conserved arginine residues of this electrostatic path [64]. This identified a specific citrate synthase mutation (R65A), which retained high catalytic activity, but the probability of channelling decreased from 0.99 to 0.023. Another intriguing feature which became apparent in these studies was that structural and kinetic analysis demonstrated that recombinant versions of malate dehydrogenase and citrate synthase self-assemble* in vitro*. Moreover, a recent study, suggests that metabolite concentration gradients enhance assembly of the TCA cycle metabolon [54]. It has also been recently demonstrated that enzymes move towards higher substrate concentrations in microfluidic devices, a phenomenon they termed collective directional diffusion [65]. Using the same microfluidic device the codiffusion of malate dehydrogenase and citrate synthase occurred against a concentration gradient of l-malate and furthermore demonstrated enzyme interactions even at very low protein concentrations. The authors interpreted their results to suggest that the OAA concentration gradient between malate dehydrogenase and citrate synthase can reduce their spatial separation and thereby increase their interactions. However, it is not clear whether such gradients are present* in vivo* and therefore whether this mechanism can regulate complex assembly, although it has long been known that metabolites of the mitochondrial matrix form isolated pools as opposed to being uniformly distributed [66].

### 2.3. Evidence for Substrate Channelling in the TCA Cycle Coming from Labelling

A variety of isotope labelling approaches have also been used to provide direct or indirect evidence for substrate channelling. For example, conserved positional transfer of label between metabolites in a pathway, where those metabolites are symmetrical, provides evidence of direct site-to-site transfer or ‘tight channelling' [67]. If symmetrical molecules are free to diffuse, then rotation of molecule leads to randomization of the position of label transfer. Absence or reduction of label randomization between equivalent atoms in symmetrical molecules therefore provides evidence for substrate channelling. In the first of these studies, rat liver mitochondria were incubated with [5-^13^C] glutamate and label distribution between the two carboxyl carbon atoms of aspartate, the reaction sequence separating these metabolites spanning the TCA cycle reactions involves the symmetrical intermediates succinate and fumarate. In their experiment, despite varying glutamate concentrations and osmotic potentials Bernhard and Tompa were unable to provide evidence that rotation was restricted suggesting either that the intermediates diffuse at random or by a direct transfer mechanism which allows rotation [68]. In the same year Sumegi and coworkers tested the same possibility in yeast driven partially by their contention that the conditions used by Bernhard and Tompa might stimulate release and subsequent uptake of intermediates by the mitochondria [69]. Sugemi et al. fed [3-^13^C] propionate to yeast cells; this substrate is sequentially converted to propionyl-Co and methylmalonyl-CoA followed by formation of [2-^13^C] succinyl-CoA before being oxidized in the TCA cycle. Therefore, the level of symmetry in the C2 and C3 position of alanine, which is linked to malate via a transamination reaction subsequent to its conversion to pyruvate by malic enzyme, was measured. Intriguingly, the labelling between these atoms was asymmetric under normal conditions, but symmetric after malonate inhibition of succinate dehydrogenase or under conditions that promoted malate to fumarate back-flux. These experiments thus provided the first evidence that substrate channelling of TCA cycle enzymes occurs* in vivo* and may be responsive to the biochemical demands on the cycle.

In other organisms, there is also evidence for tight channelling of TCA cycle intermediates, although to a variable extent. For example, although glutamate labelling studies in rat revealed asymmetric labelling [67], a more recent study in rat brain using [2-^13^C,^15^N] aspartate labelling to probe the significance of the glial-specific pyruvate carboxylase reaction and its possible involvement in a metabolon in this tissue [70], revealed less evidence of asymmetric labelling. And supply of a wide range of isotopically labelled substrates provided support for the partial channelling of the TCA cycle in the human neuronal cell line AGE1.HN [71]. By contrast, very tight channelling was observed in the urea cycle of permeabilized rat hepatocytes where even a 200-fold excess of intermediary substrates had no effect on the accumulation of ^14^C labelled urea from exogenously supplied NH_4_Cl, [^14^C]HCO^3-^ [72]. Important further support for the theory that the earlier results of Sumegi and coworkers provided evidence of channelling, came from similar experiments in which yeast were incubated in [4-^13^C]glutamate where asymmetric labelling of aspartate was observed consistent with orientation-conserved transfer of succinate and fumarate [73]. Detecting such changes* in vivo* is greatly complicated by the fact that even in the case of 100% conservation of orientation several cycles of the pathway would render labelling patterns indistinguishable from those expected from complete rotational randomization [74]. In order to circumvent this problem the authors looked for, and found, a time-dependent change in labelling symmetry [73].

Labelling approaches have also been used as a more general test of the occurrence of channelling, but without revealing the precise mechanism. For example, the ‘isotope dilution' method uses the extent of dilution of labelled metabolite pools following addition of unlabeled metabolites as a measure of substrate channelling. For example, Zhang et al. fed isolated potato tuber mitochondria with ^13^C labelled pyruvate or glutamate until accumulation of ^13^C in the downstream TCA cycle metabolites reached isotopic steady state [53]. Then unlabeled intermediates of the TCA cycle were added and the “dilution” effect on labelling was monitored over time. For the pyruvate feeding experiments, the TCA cycle was linearized by inhibiting succinate dehydrogenase with malonate and for the glutamate experiments by inhibiting aconitase with fluoroacetate, in order to prevent the complications in interpretation of labelling patterns caused by multiple turns of the cycle. These experiments revealed the dilution in 2-oxoglutarate, succinate and malate, but none in citrate or fumarate, indicating that the latter metabolites are channelled. Interestingly, the results from a metabolic flux analysis based on ^13^C-label redistribution in heterotrophic Arabidopsis cell culture indicated channelled flux from fumarate to malate, but no channelled flux from 2OG or succinate to citrate [75]. More detailed studies in potato tuber mitochondria following ^13^C-labelling revealed channelling of citrate and fumarate [53].

### 2.4. Evidence of Substrate Channelling in the TCA Cycle from Approaches Not Requiring Labelling

**Figure 2:**
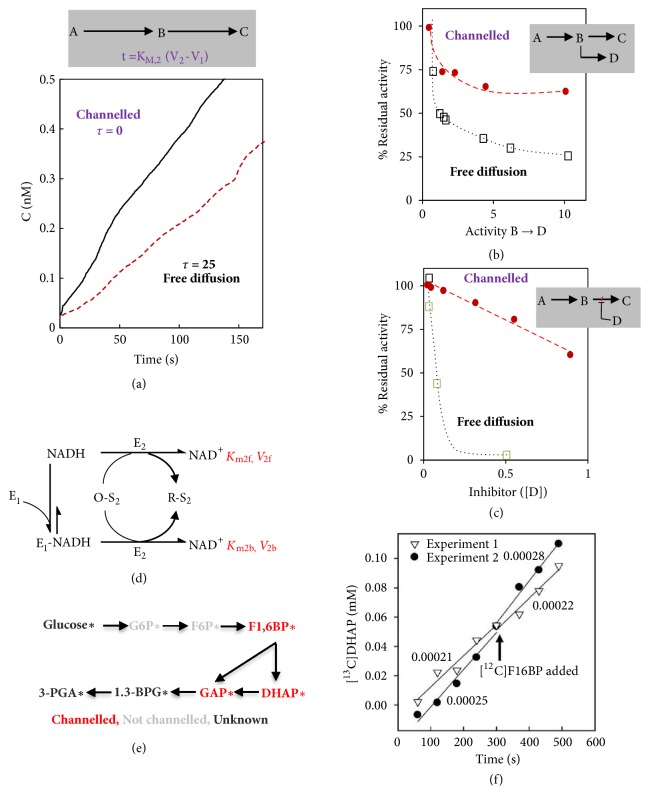
**Methods of identifying substrate channelling.** (a) A reaction scheme and depiction of transient time (t) analysis based on data from a channelled bifunctional thymidylate synthase-dihydrofolate reductase (TS-DHFR) and a freely diffusing nonfunctional TS and DHFR (data from [7]). (b) Comparison of residual activity of a channelled or freely diffusing enzyme pair in the presence of a competing enzyme, for example, the malate dehydrogenase and citrate synthase couple in the presence or absence of alanine aminotransferase which competes for the metabolic intermediate (data from [7]). (c) Comparison of residual activity of a channelled or freely diffusing enzyme pair in the presence of an inhibitor of the second enzyme, for example, the inhibition of the TS-DHFR cascade by the inhibition of DHFR by pyruvate (data from [7]). (d) Enzyme buffering analysis of channelling; this approach is typically applied for following the channelling of NADH which assesses if the second enzyme of a couple can use bound as well as free NADH and is based on comparison of the reaction velocities following dramatic decreases in the size of the free NADH pools as represented in the scheme. If the enzyme is not able to utilize bound NADH the system is essentially just buffering NADH added to it hence the name (data from [7]). (e) Schematic representation of the isotope dilution experiment to assess the channelling of glycolysis. ^13^C labelled glucose was fed to isolated potato mitochondria and the label accumulation in succinate was monitored. Nonlabelled G6P, F6P, F1,6BP, DHAP, or GAP was separately added to the medium following the fractional enrichment in succinate reaching steady state [18]. (f) The result of isotope dilution experiments for F1,6BP. The time course plots showing the fractional ^13^C enrichment in DHAP following the addition of F1,6BP at 0 min. Data come from [18].

Nonlabelled approaches of two different types have also been used to provide indirect evidence of channelling: (i) evaluation of transient times and (ii) comparison of reaction rates in the presence or absence of competing reactions or the presence or absence of an inhibitor of the second reaction. The transient time (*t*), of a sequential reaction, is the lag phase before steady state levels of each intermediate are reached and the reaction rate is constant. A simple example is presented in Figure 2(a) which provides both the underlying equations and a graphic cartoon. In a perfectly channelled reaction, t approaches zero and this value increases with increasing leakiness. This approach has been used to assess the channelling of the bifunctional TS-DHFR from* Toxoplasma gondii *[76]. While the example is for a two-step reaction it can be applied to a longer reaction cascade with the overall* t* being equal to the sum of* t*'s for each step [77]. A major limitation of this method is that it can only be applied to pathways with a measurable lag phase in the reaction time course. It also requires careful control experiments as other features of the system, such as alterations in the kinetics of the enzymes or relative concentrations of the enzymes also affect lag time. The alternative approach is to perturb substrate channelling from the bulk environment as exemplified in Figures 2(b)-2(c) which represent the perturbation by a competing enzyme or the presence of an inhibitory molecule within the bulk environment. The first approach has been used to assess channelling mediated by a malate dehydrogenase-citrate synthase couple in the presence or absence of alanine amino-transferase [78], while the second was applied to the bifunctional TS-DHFR in the presence and absence of TMP and Pyr [76]. While seemingly straightforward these analyses are highly complicated by a large number of further factors (these are detailed at length in the excellent review of [77]), which have combined to limit their utility in practice. A final nonstructural method that merits discussion is the so-called enzyme buffering method which has proven highly informative, if complex, in studies of NADH channelling by NADH dehydrogenases. In this approach, the question that is asked is whether the second enzyme (E2) can use enzyme 1 (E1) bound form of the common intermediate in addition to the free form. Given that the dissociation constant Kd of E1-NADH is usually about 1 mM with excess EI it is possible to reduce [NADH]r to a value well below its Km for E2. The larger arrow in Figure 2(d) indicates that for such a binding equilibrium NADH is more than 99% in its E1 bond form. Hence if the experimentally determined velocity is greatly in excess of that which could be achieved from the free NADH alone then this constitutes proof that the NADH is channelled between the enzymes [79]. Intriguingly, NADH channelling has only been found for enzymes of opposite chirality and clear-cut results have been reported for several enzyme pairs of opposite chirality [79]. By contrast, if NADH is not channelled then E1 is simply buffering NADH to a low [NADH]f, hence the name of the test [77]. Two further studies are of interest here. In the first NADH binding was experimentally quantified in actively respiring plant mitochondria and it was demonstrated that NADH levels were maintained at low but stable levels (Kasimova et al., 2006). In the second, a mathematical model for respiration was presented with the channelling of NADH being one of the factors considered with this study which alongside the experimental data of the Kasimova et al. study suggests that a considerable portion of NADH is protein bound rather than being freely available (Hagedorn et al., 2004). That aside, the above approach is currently only reported for NADH-associated reactions, this approach still has broad utility given the prevalence of dehydrogenase reactions in the cell.

In summary, a broad range of evidence exists for substrate channelling in the TCA cycle, the pathway around which Srere initially developed his concept of the metabolon. It is clear that channelling of TCA cycle intermediates occurs to a variable degree in different biological systems and those protein-protein interactions are prevalent amongst the constituent enzymes of the pathway.** F**urthermore strong structural support is available for the canonical malate dehydrogenase-citrate synthase-aconitase metabolon, yet evidence for channelling at other steps is only currently strongly provided for the channelling of fumarate in plants.

## 3. Glycolysis

As for the TCA cycle, the first reports of assemblies of glycolytic enzymes appeared more than 40 years ago. Initial evidence, much disputed [26, 80], came from observations of independent pools of glycolytic intermediates in* E. coli* and rats [81, 82]. Subsequently, stronger evidence for assemblies of glycolytic enzymes emerged, including direct evidence of specific interactions between sequential pairs of glycolytic enzymes, interaction of enzymes of glycolysis and actin [83, 84] or red blood cell membranes [82, 83], isolation of glycolytic particles, complexes of all glycolytic enzymes [82, 83] and electron microscopic evidence [85]. Early examples include glyceraldehyde phosphate dehydrogenase binding to microsomal fractions of skeletal muscle [86] and aldolase binding to microsomal fractions of rat liver [87]. There is evidence that glycolytic enzymes interact with red blood cell membranes [88] and interestingly that the degree of binding is environmentally dependent [89]. Likewise aldolase, glyceraldehyde phosphate dehydrogenase, lactate dehydrogenase, and pyruvate kinase were demonstrated to bind to columns onto which the F-actin- tropomyosin complex had been bound [90]. A particular breakthrough in establishing the occurrence of glycolytic enzyme assemblies was observations of specific interactions between sequential enzymes with glyceraldehyde phosphate dehydrogenase and aldolase being a recurrently observed pairing [28]. These interactions were revealed by a number of experimental approaches including kinetic studies, polarization of fluorescence, affinity electrophoresis [39], and cocentrifugation of sequential enzyme activities. Similar observations have been made for aldolase and triose phosphate isomerase [91], glyceraldehyde phosphate dehydrogenase and phosphoglycerate kinase [92], and the entire subpathway operating between fructose-1, 6-bisphosphate, and phosphoenolpyruvate [93]. However, simple gel filtration of cell extracts of* E. coli* [94] and* S. cerevisiae* [28] revealed only a small proportion of the enzymes to be in complex with one another. Additionally, phosphorylation of phosphofructokinase alters not only its kinetic behavior, but also its binding to actin [95]. There is also a large body of evidence that suggests interaction of glycolytic enzymes with mitochondria. For example, a study of* Tetrahymena pyriformis* revealed that 100% of lactate dehydrogenase, 75% of phosphofructokinase, and 50% of glyceraldehyde phosphate dehydrogenase are bound to mitochondria [88–90], while binding of hexokinase to the mitochondrial membrane was recognized early to be a common feature of eukaryotes [28]. It was postulated that this location of hexokinase was advantageous in terms of energy efficiency, providing ready access to the ATP being generated by the mitochondria [28]. Also relevant is the intriguing presence in trypanosomes of the glycosome, a specialized organelle that contains all of the glycolytic enzymes [96]. A potential explanation for this is that this organism generates all its ATP via glycolysis. Notably, the trypanosome glycolytic enzymes can be isolated as a complex following the removal of the organellar membrane leading to the suggestion that metabolic channelling occurs, with associated kinetic and regulatory benefits [28]. We will provide a critical analysis of this concept later in the article.

### 3.1. Application of Modern Systems-Based Approaches to Study Enzyme Assemblies of Glycolysis

**Figure 3:**
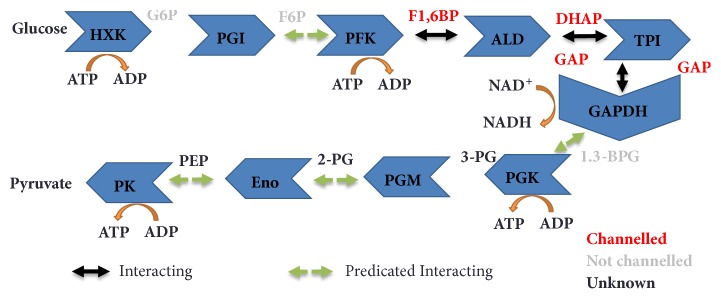
**The substrate channel and protein interaction of the glycolysis. **The plant protein interactions of catalytic subunits that potentially mediate metabolite channelling are described next to the arrows. Metabolites drawn as red, grey, and black text are channelled, not channelled, and not tested, respectively [18, 56, 57]. GAP, glyceraldehyde-3-phosphates; G6P, glucose-6-phosphate; F6P, fructose-6-phosphate; F1,6-BP, fructose-1,6-bisphosphate; DHAP, dihydroxyacetone phosphate; GAP, glyceraldehyde 3-phosphate; 1,3-BPG, 1,3-bisphosphoglycerate; 3PG, 3-phosphoglycerate; 2PG, 2-phosphoglycerate; PEP, phosphoenolpyruvate; HXK, hexokinase; PGI, phosphoglucose isomerase; PFK, phosphofructokinase; ALD, aldolase; TPI, triosephosphate isomerase; GAPDH, glyceraldehyde phosphate dehydrogenase; PGK, phosphoglycerate kinase; ENO, Enolase; PK, pyruvate kinase.

When taken together the studies described above already provide relatively strong evidence for glycolytic complex formation but this has been significantly bolstered by a wealth of cross-kingdom evidence this century [7, 56]. A handful of proteomic studies suggested the presence of the enzymes of glycolysis in isolated mitochondrial fractions from Arabidopsis [57], humans [97], and yeast [98] (Figures 2(e), 2(f), and 3). Studies in yeast employing both enzyme assays and blue native SDS-PAGE and coimmunoprecipitation of proteins with anti-enolase antibodies revealed that enolase takes part in a large macromolecular complex associated with the mitochondria and including mitochondrial membrane carriers and enzymes of the TCA cycle [99]. In addition, they found an unsuspected novel function of this complex in the mitochondrial import of tRNA [99]. In the Arabidopsis study, a combination of protease treatments and cell biology techniques was used to demonstrate that these enzymes were mainly localized on the cytosolic face of the outer mitochondrial membrane; i.e., they were attached to the surface of the organelle [57]. Experiments in which the glycolytic substrates ^13^C-glucose and 1-^13^C fructose-1, 6-bisphosphate were supplied to isolated mitochondria demonstrated that the complete glycolytic sequence was present and active in this fraction. Subsequent studies from the same group, but this time working on the far more experimentally tractable potato mitochondria, proved the dynamics of this association. Intriguingly, inhibition of respiration by KCN led to a proportional decrease in association of glycolytic enzymes with mitochondria and conversely stimulation of respiration by a range of means enhanced the association which appears to be mediated by the outer mitochondrial membrane protein VDAC anchoring the glycolytic enzymes to the membrane [18]. Importantly, this study also provided indirect evidence for the channelling of the glycolytic intermediates with the labelling patterns of glycolytic intermediates being followed by NMR suggesting a leaky channelling of glucose 6-phosphate and fructose 6-phosphate but a tighter channelling of intermediates from fructose 1,6-bisphosphate onwards [18]. Channelling in mammalian glycolysis is likely less tight than that found in plants [100]. Channelling within glycolysis also appears to occur in organisms such as* E. coli*, which lack mitochondria [101].

### 3.2. Analysis of Isotopic Labelling and NADH Channelling in Glycolysis

Two approaches that were utilized for the TCA cycle and described above have proven similarly useful in assessing the presence of metabolite channelling in glycolysis, namely, analysis of NADH channelling and modelling of ^13^C isotopic labelling studies (Figures 2(e) and 2(f)). The first of these was an early experiment by Srivastava and Bernhard which demonstrated the direct transfer of NADH from liver glyceraldehyde phosphate dehydrogenase to alcohol dehydrogenase [102]. Further work from this group has demonstrated that a series of such transfers can occur between dehydrogenases and that in every case the transfer occurs between dehydrogenases with opposite stereospecificity for NADH. Moreover, computer-based analysis of the electrostatic potential of this enzyme pair interaction suggests that the active site region of the glyceraldehyde phosphate dehydrogenase is positive while that of the alcohol dehydrogenase is negative [103]. These considerations, as well as the suggestion made by Paul Srere that a constancy of size is required between the interacting enzymes [28], give some insight into the complex selective pressures apparent during the evolution of such interactions. The second approach was additionally able to demonstrate that modelling data obtained from isotope labelling experiments in a manner which includes channelled glycolysis provides a better fit to the data than when channelling of this pathway is not considered [79, 104].

There are six further publications regarding glycolytic assemblies that we feel merit discussion here: a review on the activity of glycolytic metabolons in muscle [56], a study of the glycolysis actin interaction in yeast [98], three cell biology studies on glycolytic assemblies in animals [97, 105, 106], and finally an example of chemotactic driven enzyme assembly using glycolytic enzymes and intermediates in a cell-free system [60]. The review on muscle presents a comprehensive overview of our understanding of muscle glycolysis making the interesting point that one factor driving the formation of a glycolytic metabolon may be the fact that the protein concentration of the cytosol is close to that at which protein crystals form such that a presence of metabolons may increase the solvation capacity of the cell [56]. The authors go on to define three commonly observed subcomplexes in muscle (i) PFK, aldolase and glyceraldehyde phosphate dehydrogenase, (ii) triose phosphate isomerase and phosphoglycerate kinase, and (iii) phosphoglucomutase, enolase, and pyruvate kinase. Indeed these subcomplexes appear largely to associate with one another in a ratio other than the simple 1:1:1 ratio which would be anticipated to be optimally efficient. However, despite a wealth of binary interaction data the authors concluded that there was not enough data to currently unequivocally establish the structure of the glycolytic metabolon yet in muscles. A similar conclusion was reached following the study of the role of F-actin in yeast [98]. However, here the authors did clearly demonstrate the importance of F-actin in stabilizing the glycolytic assembly and showed that actin-associated enzymes were able to maintain high activities in the presence of high concentrations of compatible solutes than the nonassociated enzymes [98]. In the same year the label transfer method by which the photoactivatable cross linkers reacted with glycolytic enzymes which subsequently transfer the label to their binding partners provided further strong evidence of association amongst the glycolytic enzymes [97]. This technique revealed known binding partners as well as the novel partners a- and b-spectrin, ankyrin, p55, and protein 4.2. This study also provided molecular details on the specific proteins which form the interfacial contacts within each interaction. Finally, these complexes of glycolytic enzymes could be spatially localized to areas on the membrane where ATP was rapidly consumed [97]. More recently an elegant cell biology approach has also been used to demonstrate that glycolytic assemblies occur at* C. elegans* neuronal synapses during energy stress and are indeed required to maintain vesicle protein clusters during these phenomena and as such to be essential in the synaptic vesicle cycle [105]. Fluorescence microscopy techniques such as FRET and FRAP have additionally recently been used to observe the dynamic formation of glycolytic assemblies in living human cells [106]. A final fascinating recent finding of note is the fact that using microfluidic and fluorescent spectroscopic techniques Zhao et al. recently demonstrated that the first four enzymes of the glycolytic pathway each independently follows its own specific substrate gradient, providing interesting insight into the potential mechanism of the assembly of enzyme complexes [60].

In summary, there is a vast wealth of evidence that glycolytic assemblies are present in all three kingdoms of life, even if their constitution may vary somewhat. Recent proteomic and molecular cell biology approaches have largely substantiated historical biochemical evidence of these assemblies. However, functional proof of substrate channelling remains relatively rare and even quantification of the extent of channelled versus unchannelled events is lacking. In order to better understand the roles of these assemblies more precise techniques that will enable insight into the mechanism of the channelling will be required. Nevertheless, great steps have been made in approaching the biological function of glycolytic enzyme assemblies within the last two decades and at least in plants there is strong proof for substrate channelling (Figure 3).

## 4. The Mitochondrial Electron Transport Chain

### 4.1. The Presence of Respiratory Supercomplexes

**Figure 4:**
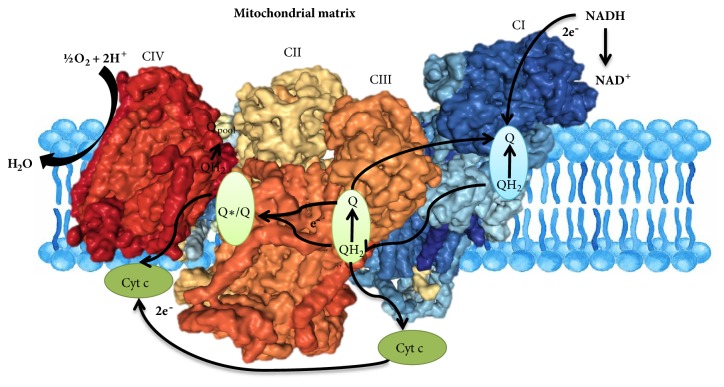
**The supercomplexes of the mitochondrial electron transport chain complexes (modified from [58]). **Two electrons from NADH (top right) are passed through complex C I (blue), reducing Q to QH2. Diffusing from the Q-tunnel, QH2 may enter the proximal Q cavity of C III (the oxidation cavity) or may diffuse into the membrane pool. QH2 passes one electron to cyt c in the intermembrane space and one to Q in the distal cavity (the reduction cavity), creating the Q• intermediate. Oxidation of a second QH2 in the proximal cavity will lead to reduction of Q• in the distal cavity to QH2, which can then leave and join the membrane pool. CI, Complex I (NADH:ubiquinone oxidoreductase); CII, Complex II (succinate dehydrogenase); CIII, Complex III (cytochrome bc1); CIV, Complex IV (cytochrome c oxidase); cyt c, cytochrome c.

The respiratory complexes are well defined stable multisubunit complexes that have been subject to considerable research effort for many reasons including the importance of respiratory chain dysfunction in aging and disease [22]. The individual respiratory complexes can be dynamically organized into supercomplexes and these have been observed in mammals [107, 108], plants [109, 110], yeast [107], and even some bacteria such as* Paracoccus denitrificans* [111] (Supplemental [Sec supplementary-material-1]). However, some peculiarities have been reported. For example,* E. coli* contains patches of identical complexes in different parts of its cell membrane [112], while BN-PAGE has revealed supercomplexes of varying stoichiometries with Complexes I and III being far more likely to be found in a supercomplex than Complex IV [108]. Moreover, in* S. cerevisiae, *which does not express Complex I, only simpler super-assemblies of Complexes III and IV are present [113, 114]. Despite initial skepticism, respiratory supercomplexes are now accepted by the scientific community. However, the reasons for their existence and the question as to whether they confer any functional advantage remain under considerable debate [22]. Indeed, by contrast to the TCA cycle and glycolytic pathways described above, evidence that the supercomplexes perform substrate channelling remains hotly contended with groups debating whether structural evidence is consistent with the occurrence of this phenomenon or not [115–118]. In this section, we will critically review the evidence for substrate channelling within respiratory supercomplexes, as well as suggesting experimental strategies which may provide more conclusive answers. To be clear, in this section we are only discussing channelling between respiratory complexes and not whether it occurs between the reaction centers within the respiratory complexes themselves (Figure 4).

### 4.2. Are Respiratory Supercomplexes Carrying out Substrate Channelling?

There is a plethora of structural evidence for the existence of respiratory supercomplexes so we will start by highlighting the key findings of these studies and their implications. While these supercomplexes were initially dismissed as artefacts of mild detergent solubilization, they were subsequently observed in the absence of detergent and are now widely accepted [22]. This is in part due to the fact that developments in single-particle electron cryomicroscopy has led to relatively high-resolution of the mammalian respirasomes (supercomplexes that are capable of NADH: O_2_ oxidoreduction* in vitro*). The structure of the porcine respirasome and three structures from bovine heart muscle (two respirasomes and one lacking Complex IV) [116, 118–120] revealed a conserved arrangement of Complexes I and III, but varying location of Complex IV. These supercomplexes may vary further in structure but may also be subject to dynamic disassembly/assembly and reorganization. Alternatively, this variance in composition could be due to different approaches in particle classification adopted by the authors [22]. Irrespective of these differences, these structures provided new insights concerning the interactions between subunits of the different complexes. Interactions between Complexes I and III are primarily mediated by two regions, NDUFA11 and NDUFB4, of Complex I that interact with UQCRQ of Complex III and NDUFB9 and NDUFB4 of Complex I that interact with UQCRC1 and the Rieske protein [22]. Interactions between Complex IV and Complexes I and III vary between the different respirasome structures [118, 120]. Two small protein families have been reported to have assembly and/or stabilizing functions with regard to the supercomplexes. The first of these proteins, the respiratory complex factor family [121], most probably acts via an indirect mechanism so it is perhaps not surprising that no densities of any of its homologs are observed in current structural models [22]. By contrast, supercomplex assembly factor was described as a specific factor in the formation/ stabilization of respirasomes [122] and a range of detailed mutagenic studies showed that this protein and its homologs have important roles in supercomplex assemblies [58], while densities attributable to this protein have been found within recent structural models [22] (Figure 4). Furthermore, the lack of a plant homolog of this protein may explain the absence of Complex IV in the Asparagus supercomplex structure [123].

Despite the unprecedented structural information, it remains difficult to derive information about the catalytic mechanisms of redox enzymes from their structures [22]. This fact probably explains how the same structural evidence was interpreted to both support and refute the likely occurrence of substrate channelling. Kühlbrandt and coworkers suggested that the supercomplexes form solid-state devices facilitating enclosed exchange of ubiquinone/ubiquinol between Complexes I and III and for cyt c between Complexes III and IV [115, 116]. By contrast, Boekema and coworkers noted that, while the proximity of active sites may reduce random diffusion time, they did not feel this was likely the reason for supercomplex assembly [117]. Sazanov and coworkers went even further stating that there is a lack of any substrate channels, or barriers to free diffusion, to connect the substrate binding sites of Complexes I, III and IV within their structures [118]. Indeed, despite a range of other postulated channels (reviewed in [22] and requiring as yet unobserved catalytic mechanisms of the complexes), the current consensus from structural studies appears to lean towards an absence of channelling [22, 58, 118, 124].

The amount of functional or physiological evidence available in support of channelling between the supercomplexes is even less than that from structural studies. And much of it is rather circumstantial. For example, the fact that these complexes form under conditions of stress which presumably require more efficient energy production has commonly been stated as support for substrate channelling within the respiratory supercomplexes. Certainly there is good cross-kingdom support for variance in the composition and abundance of respiratory chain supercomplexes depending on metabolic and physiological conditions [107, 110, 125–129], in addition to the lipid composition of the mitochondrial inner membrane [130, 131]. In addition to these phenomenological observations certain other physiological measurements have been interpreted as evidence for substrate channelling of coenzyme Q. For example, results from flux control analysis of respiration has been interpreted to provide evidence of respirasomes [132], subsequent analysis has shown that the results of such studies are highly dependent on the respiratory inhibitor [133] used, and the high extrinsic concentrations of hydroquinones and cyt c used to assay the individual reactions has also been queried [22]. Similarly, fusion of submitochondrial particles with liposomes, effectively diluting the respiratory complexes, followed by measuring the rate of respiration was also argued to provide proof of channelling [134]. That said more convincing evidence has been provided that the supercomplexes define dedicated coenzyme Q and cytochrome* c* pools [125] with each supercomplex containing its own tiny pool of each which do not exchange with the similarly tiny pools of other supercomplexes. However, the data can equally be explained by alternative phenomena, for example, the fact that fibroblasts with low Complex III expression were characterized with decreased Complex I but increased Complex II activities were subsequently explained by increased ROS production leading to phosphorylation mediated activation of Complex II [135] and Complex I disassembly [136], respectively. As mentioned above, the studies of Blaza and coworkers also provided no evidence in support of channelling [133]}. Recently, a definitive experimental test for the presence of channelling of coenzyme Q between respiratory complexes was completed and this strongly suggests that channelling does not occur [137]. The test involved the introduction of a competing enzyme, in this case recombinant trypanosome alternative oxidase (AOX) into the electron transport chain of isolated submitochondrial particles from mammalian heart. If coenzyme Q is completely channelled between Complex I and Complex III then there will be little flux through the AOX because it will not have access to its substrate. The opposite was found: addition of AOX increased the rate of oxidation of NADH 4-fold and this increased rate of flux through the coenzyme Q pool was sensitive to inhibitors of AOX. It was further shown that addition of AOX did not disrupt supercomplex formation.

Thus all things considered we are of the opinion that robust evidence for the channelling of electron carriers between respiratory complexes is lacking. If correct, this begs the question of the physiological purpose of supercomplex formation. Alternative suggestions include stabilization of the individual complexes, regulation of respiratory chain activity, modulation of cristae morphology, and the prevention of protein aggregation in the extremely protein-rich mitochondrial membrane [22].

## 5. Future Perspective

The concept of multienzyme assemblies that promote substrate channelling has gradually gained credence over the last four decades, from initially being regarded as a biochemical curiosity to now being appreciated as a widespread phenomenon in nature with rich potential for exploitation in synthetic biology [7, 38]. However, while there is accumulating evidence for enzyme-enzyme interactions and indeed structural analyses of the resulting complexes, direct tests of substrate channelling remain rare and the functional significance of the assemblies remains elusive. The fact that the formation of enzyme assemblies within the respiratory pathways is condition-dependent strongly suggests that the phenomenon has regulatory significance, most likely by control of flux at branch points with competing pathways. But as the case with the respiratory chain supercomplexes highlights, there can be other functional reasons for enzyme-enzyme assemblies that may have more to do with local cell biological structures and biophysics. The recent development of super-resolution microscopy techniques [138] and technological developments for the study of metabolite-protein interactions [139, 140] have great potential for further characterizing the functional significance of enzyme assemblies. And synthetic biology, by creating enzyme assemblies and assessing their consequence in host cells or synthetic proto-cells, is also likely to provide further insight into their functional properties.

## Supplementary Materials

Supplementary Materials
** Supplementary Figure 1**.** Components of tricarboxylic acid (TCA) cycle. **Schematic summary of the TCA cycle and the enzymes related to the reactions [53]. The large text and the arrows represent the intermediates and reactions, respectively. The enzyme names are shown in the blue rectangles. PDC, pyruvate dehydrogenase complex; ME, malic enzyme; CSY, citrate synthase; ACO, aconitase; IDH, isocitrate; ODC, oxoglutarate dehydrogenase complex; SCoAL, succinyl-CoA ligase; SDH, succinate dehydrogenase; FUM, fumarase; MDH, malate dehydrogense; 2OG, 2-oxoglutarate.** Supplementary Figure 2. The pathway of the glycolysis. **Abbreviations: GAP, glyceraldehyde-3-phosphates; G6P, glucose-6-phosphate; F6P, fructose-6-phosphate; F1,6-BP, Fructose-1,6-Bisphosphate; DHAP, dihydroxyacetone phosphate; GAP, glyceraldehyde 3-phosphate; 1,3-BPG, 1,3-bisphosphoglycerate; 3PG, 3-phosphoglycerate; 2PG, 2-phosphoglycerate; PEP, phosphoenolpyruvate; HXK, hexokinase; PGI, phosphoglucose isomerase; PFK, phosphofructokinase; ALD, aldolase; TPI, triosephosphate isomerase; GAPDH, glyceraldehyde phosphate dehydrogenase; PGK, phosphoglycerate kinase; ENO, enolase; PK, pyruvate kinase.** Supplementary Figure 3. Schematic of mitochondrial electron transport chain. **The electron transport chain is a series of electron transporters embedded in the inner mitochondrial membrane that shuttles electrons from NADH and FADH2 to molecular oxygen. In the process, protons are pumped from the mitochondrial matrix to the intermembrane space, and oxygen is reduced to form water. Abbreviations: CI, Complex I (NADH:ubiquinone oxidoreductase); CII, Complex II (succinate dehydrogenase); CIII, Complex III (cytochrome bc1); CIV, Complex IV (cytochrome c oxidase); cyt c, cytochrome c.
