# Supplementary material for: Passing the Baton: Substrate Channelling in Respiratory Metabolism
Source: Research (Wash D C). 2018 Nov 21;2018:1539325. doi: 10.1155/2018/1539325 (PMC6750097; doi:10.1155/2018/1539325)
Supplement: Supplementary Materials — Supplementary Figure 1. Components of tricarboxylic acid (TCA) cycle. Schematic summary of the TCA cycle and the enzymes related to the reactions [53]. The large text and the arrows represent the intermediates and reactions, respectively. The enzyme names are shown in the blue rectangles. PDC, pyruvate dehydrogenase complex; ME, malic enzyme; CSY, citrate synthase; ACO, aconitase; IDH, isocitrate; ODC, oxoglutarate dehydrogenase complex; SCoAL, succinyl-CoA ligase; SDH, succinate dehydrogenase; FUM, fumarase; MDH, malate dehydrogense; 2OG, 2-oxoglutarate. Supplementary Figure 2. The pathway of the glycolysis. Abbreviations: GAP, glyceraldehyde-3-phosphates; G6P, glucose-6-phosphate; F6P, fructose-6-phosphate; F1,6-BP, Fructose-1,6-Bisphosphate; DHAP, dihydroxyacetone phosphate; GAP, glyceraldehyde 3-phosphate; 1,3-BPG, 1,3-bisphosphoglycerate; 3PG, 3-phosphoglycerate; 2PG, 2-phosphoglycerate; PEP, phosphoenolpyruvate; HXK, hexokinase; PGI, phosphoglucose isomerase; PFK, phosphofructokinase; ALD, aldolase; TPI, triosephosphate isomerase; GAPDH, glyceraldehyde phosphate dehydrogenase; PGK, phosphoglycerate kinase; ENO, enolase; PK, pyruvate kinase. Supplementary Figure 3. Schematic of mitochondrial electron transport chain. The electron transport chain is a series of electron transporters embedded in the inner mitochondrial membrane that shuttles electrons from NADH and FADH2 to molecular oxygen. In the process, protons are pumped from the mitochondrial matrix to the intermembrane space, and oxygen is reduced to form water. Abbreviations: CI, Complex I (NADH:ubiquinone oxidoreductase); CII, Complex II (succinate dehydrogenase); CIII, Complex III (cytochrome bc1); CIV, Complex IV (cytochrome c oxidase); cyt c, cytochrome c. [file 1539325.f1.pptx]

## Slide 1
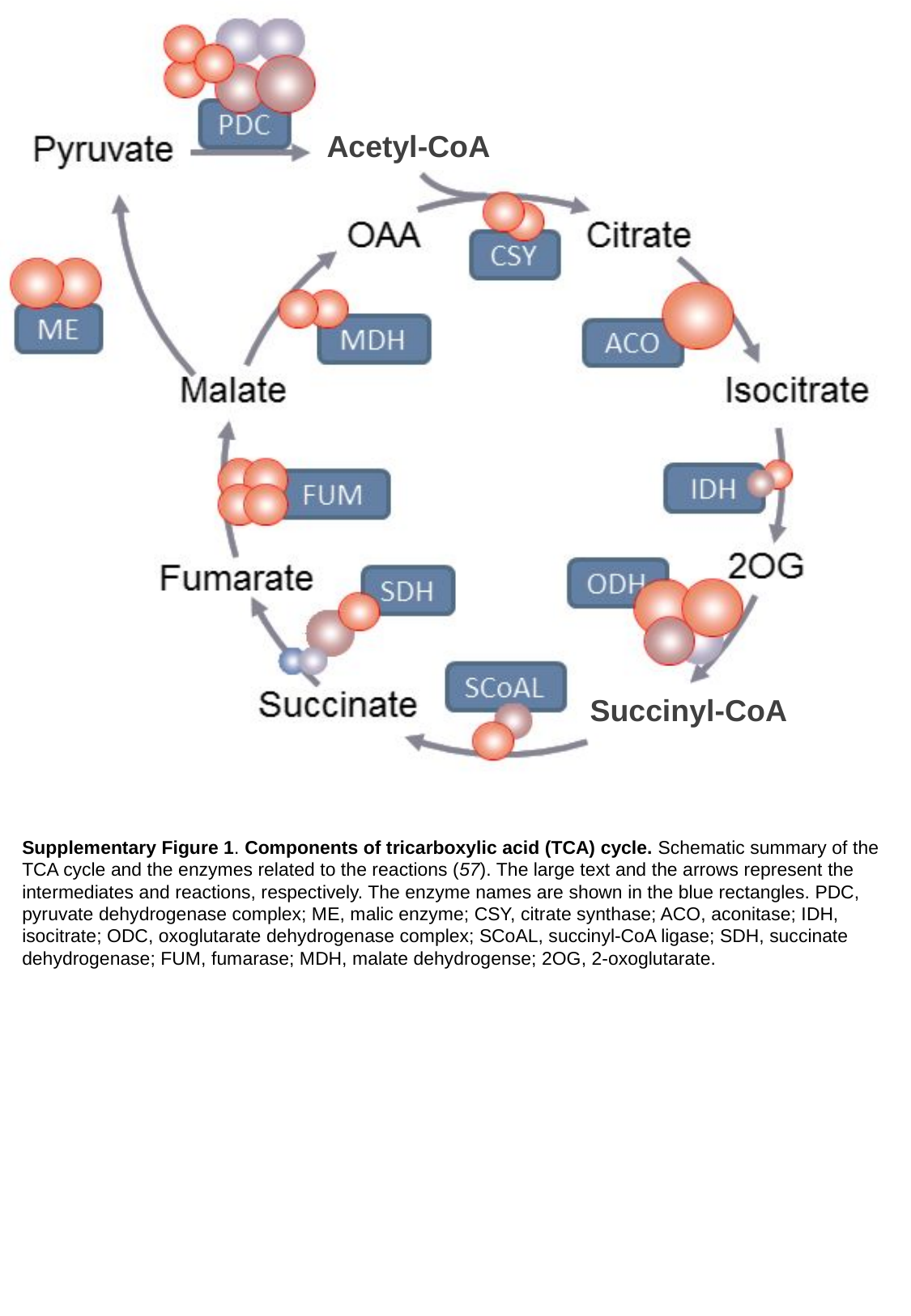

Acetyl-CoA
Succinyl-CoA
Supplementary Figure 1. Components of tricarboxylic acid (TCA) cycle. Schematic summary of the TCA cycle and the enzymes related to the reactions (57). The large text and the arrows represent the intermediates and reactions, respectively. The enzyme names are shown in the blue rectangles. PDC, pyruvate dehydrogenase complex; ME, malic enzyme; CSY, citrate synthase; ACO, aconitase; IDH, isocitrate; ODC, oxoglutarate dehydrogenase complex; SCoAL, succinyl-CoA ligase; SDH, succinate dehydrogenase; FUM, fumarase; MDH, malate dehydrogense; 2OG, 2-oxoglutarate.

## Slide 2
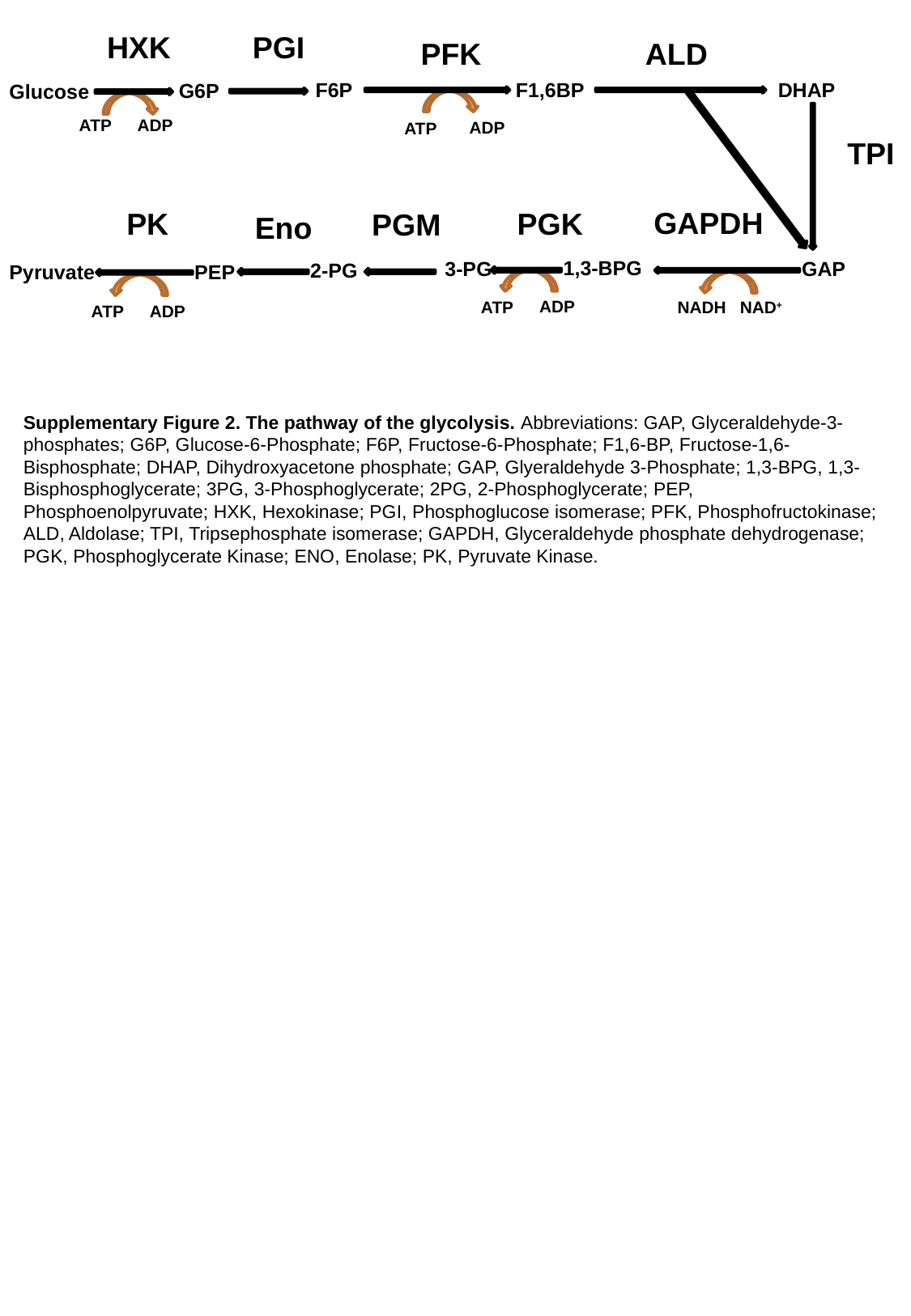

HXK
PGI
PFK
ALD
DHAP
F1,6BP
F6P
G6P
Glucose
ADP
ATP
ADP
ATP
TPI
GAPDH
PK
PGK
PGM
Eno
1,3-BPG
GAP
3-PG
2-PG
Pyruvate
PEP
ADP
ATP
NADH
NAD+
ADP
ATP
Supplementary Figure 2. The pathway of the glycolysis. Abbreviations: GAP, Glyceraldehyde-3-phosphates; G6P, Glucose-6-Phosphate; F6P, Fructose-6-Phosphate; F1,6-BP, Fructose-1,6-Bisphosphate; DHAP, Dihydroxyacetone phosphate; GAP, Glyeraldehyde 3-Phosphate; 1,3-BPG, 1,3-Bisphosphoglycerate; 3PG, 3-Phosphoglycerate; 2PG, 2-Phosphoglycerate; PEP, Phosphoenolpyruvate; HXK, Hexokinase; PGI, Phosphoglucose isomerase; PFK, Phosphofructokinase; ALD, Aldolase; TPI, Tripsephosphate isomerase; GAPDH, Glyceraldehyde phosphate dehydrogenase; PGK, Phosphoglycerate Kinase; ENO, Enolase; PK, Pyruvate Kinase.

## Slide 3
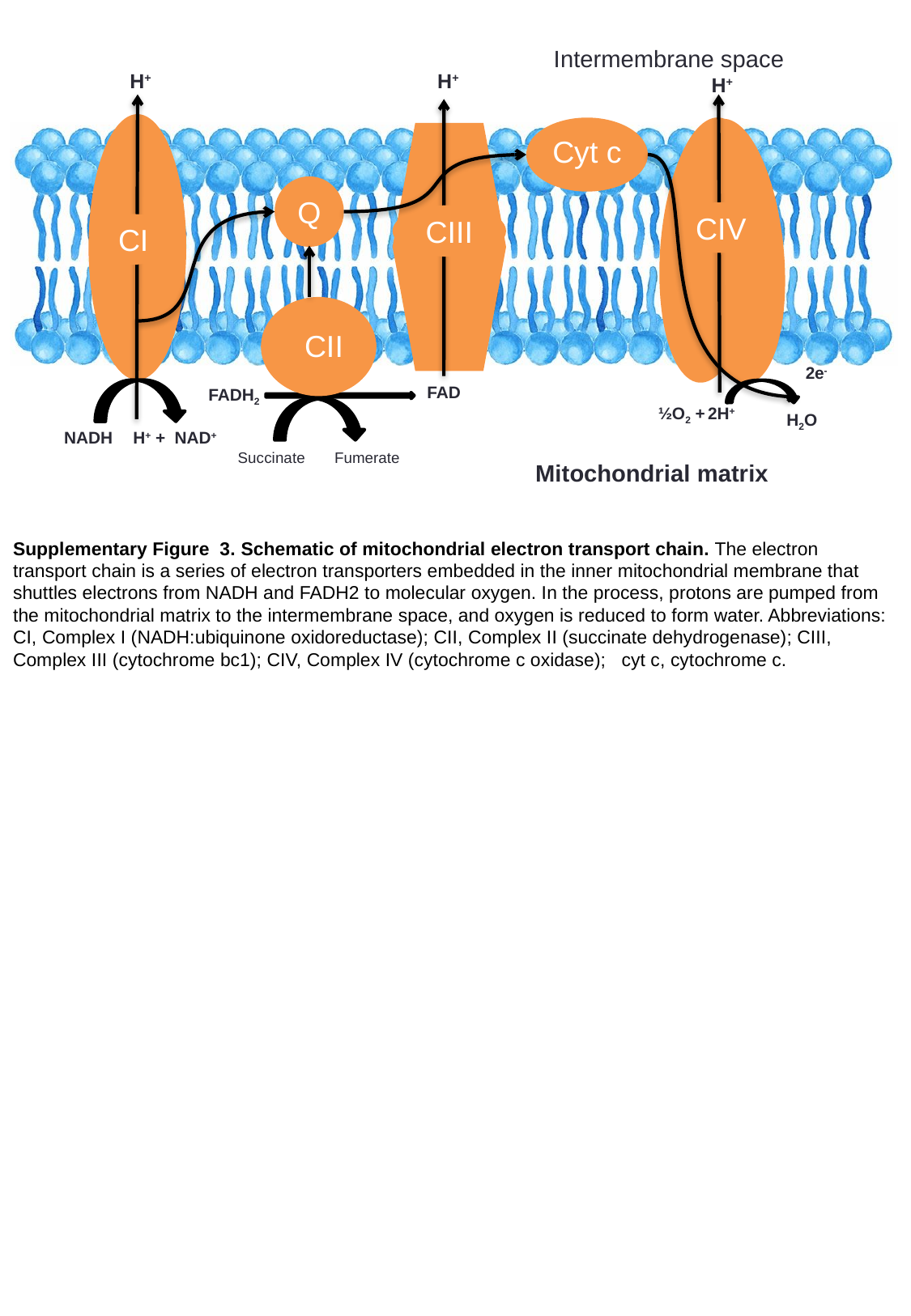

Intermembrane space
H+
H+
H+
Cyt c
Q
CIV
CIII
CI
III
CII
2e-
FAD
FADH2
½O2 + 2H+
H2O
NADH
H+ + NAD+
Succinate
Fumerate
Mitochondrial matrix
Supplementary Figure  3. Schematic of mitochondrial electron transport chain. The electron transport chain is a series of electron transporters embedded in the inner mitochondrial membrane that shuttles electrons from NADH and FADH2 to molecular oxygen. In the process, protons are pumped from the mitochondrial matrix to the intermembrane space, and oxygen is reduced to form water. Abbreviations: CI, Complex I (NADH:ubiquinone oxidoreductase); CII, Complex II (succinate dehydrogenase); CIII, Complex III (cytochrome bc1); CIV, Complex IV (cytochrome c oxidase);   cyt c, cytochrome c.
